# Supplementary material for: Role and mechanism of NCAPD3 in promoting malignant behaviors in gastric cancer
Source: Front Pharmacol. 2024 Apr 22;15:1341039. doi: 10.3389/fphar.2024.1341039 (PMC11070777; doi:10.3389/fphar.2024.1341039)
Supplement: Supplementary file 11 [file DataSheet2.ZIP › GSEA/GO_CC/my_analysis.Gsea.1599461408869/GO_CELL_SURFACE.html]

Details for gene set GO\_CELL\_SURFACE[GSEA]

|  || Dataset | filtered\_dataset.sample\_info.cls#WT\_versus\_NCAPD3\_MUT |
| Phenotype | sample\_info.cls#WT\_versus\_NCAPD3\_MUT |
| Upregulated in class | NCAPD3\_MUT |
| GeneSet | GO\_CELL\_SURFACE |
| Enrichment Score (ES) | -0.35153452 |
| Normalized Enrichment Score (NES) | -2.385041 |
| Nominal p-value | 0.0 |
| FDR q-value | 0.00666414 |
| FWER p-Value | 0.005 |
Table: GSEA Results Summary

  

Fig 1: Enrichment plot: GO\_CELL\_SURFACE      
 Profile of the Running ES Score & Positions of GeneSet Members on the Rank Ordered List

  

| SYMBOL | TITLE | RANK IN GENE LIST | RANK METRIC SCORE | RUNNING ES | CORE ENRICHMENT || 1 | 659 | BMPR2 | 16 | 1.047 | 0.0200 | No |
| 2 | 2243 | FGA | 18 | 1.039 | 0.0509 | No |
| 3 | 7976 | FZD3 | 88 | 0.815 | 0.0247 | No |
| 4 | 81619 | TSPAN14 | 212 | 0.666 | -0.0462 | No |
| 5 | 2244 | FGB | 321 | 0.582 | -0.1084 | No |
| 6 | 960 | CD44 | 339 | 0.573 | -0.1036 | No |
| 7 | 1080 | CFTR | 366 | 0.557 | -0.1059 | No |
| 8 | 6507 | SLC1A3 | 464 | 0.496 | -0.1626 | No |
| 9 | 1755 | DMBT1 | 480 | 0.487 | -0.1589 | No |
| 10 | 5245 | PHB | 481 | 0.486 | -0.1441 | No |
| 11 | 7099 | TLR4 | 541 | 0.451 | -0.1741 | No |
| 12 | 57493 | HEG1 | 683 | 0.386 | -0.2667 | No |
| 13 | 3323 | HSP90AA4P | 793 | 0.329 | -0.3375 | No |
| 14 | 5898 | RALA | 813 | 0.309 | -0.3421 | Yes |
| 15 | 1605 | DAG1 | 823 | 0.304 | -0.3395 | Yes |
| 16 | 7037 | TFRC | 824 | 0.303 | -0.3303 | Yes |
| 17 | 3146 | HMGB1 | 831 | 0.295 | -0.3257 | Yes |
| 18 | 3673 | ITGA2 | 858 | -0.264 | -0.3370 | Yes |
| 19 | 6385 | SDC4 | 871 | -0.282 | -0.3372 | Yes |
| 20 | 3309 | HSPA5 | 876 | -0.288 | -0.3314 | Yes |
| 21 | 6520 | SLC3A2 | 878 | -0.290 | -0.3233 | Yes |
| 22 | 8140 | SLC7A5 | 890 | -0.305 | -0.3222 | Yes |
| 23 | 4008 | LMO7 | 909 | -0.335 | -0.3253 | Yes |
| 24 | 301 | ANXA1 | 914 | -0.342 | -0.3178 | Yes |
| 25 | 10232 | MSLN | 925 | -0.351 | -0.3145 | Yes |
| 26 | 3691 | ITGB4 | 926 | -0.352 | -0.3038 | Yes |
| 27 | 3949 | LDLR | 929 | -0.353 | -0.2945 | Yes |
| 28 | 9388 | LIPG | 937 | -0.361 | -0.2887 | Yes |
| 29 | 5329 | PLAUR | 952 | -0.373 | -0.2878 | Yes |
| 30 | 4680 | CEACAM6 | 955 | -0.374 | -0.2778 | Yes |
| 31 | 5621 | PRNP | 970 | -0.387 | -0.2764 | Yes |
| 32 | 9180 | OSMR | 981 | -0.394 | -0.2718 | Yes |
| 33 | 9308 | CD83 | 1025 | -0.423 | -0.2908 | Yes |
| 34 | 966 | CD59 | 1040 | -0.436 | -0.2879 | Yes |
| 35 | 51704 | GPRC5B | 1043 | -0.438 | -0.2760 | Yes |
| 36 | 7132 | TNFRSF1A | 1055 | -0.449 | -0.2705 | Yes |
| 37 | 1191 | CLU | 1056 | -0.450 | -0.2568 | Yes |
| 38 | 81502 | HM13 | 1057 | -0.450 | -0.2430 | Yes |
| 39 | 1604 | CD55 | 1090 | -0.468 | -0.2525 | Yes |
| 40 | 1889 | ECE1 | 1091 | -0.468 | -0.2382 | Yes |
| 41 | 3134 | HLA-F | 1139 | -0.506 | -0.2576 | Yes |
| 42 | 2152 | F3 | 1188 | -0.559 | -0.2761 | Yes |
| 43 | 5154 | PDGFA | 1191 | -0.566 | -0.2604 | Yes |
| 44 | 23657 | SLC7A11 | 1205 | -0.581 | -0.2523 | Yes |
| 45 | 5795 | PTPRJ | 1210 | -0.586 | -0.2374 | Yes |
| 46 | 2043 | EPHA4 | 1228 | -0.602 | -0.2317 | Yes |
| 47 | 7057 | THBS1 | 1242 | -0.620 | -0.2224 | Yes |
| 48 | 1956 | EGFR | 1266 | -0.657 | -0.2194 | Yes |
| 49 | 154064 | RAET1L | 1276 | -0.664 | -0.2058 | Yes |
| 50 | 80328 | ULBP2 | 1277 | -0.664 | -0.1856 | Yes |
| 51 | 353091 | RAET1G | 1278 | -0.664 | -0.1654 | Yes |
| 52 | 4240 | MFGE8 | 1280 | -0.668 | -0.1457 | Yes |
| 53 | 5046 | PCSK6 | 1297 | -0.699 | -0.1363 | Yes |
| 54 | 1839 | HBEGF | 1328 | -0.751 | -0.1357 | Yes |
| 55 | 6509 | SLC1A4 | 1330 | -0.752 | -0.1135 | Yes |
| 56 | 7422 | VEGFA | 1346 | -0.788 | -0.1006 | Yes |
| 57 | 4233 | MET | 1358 | -0.821 | -0.0837 | Yes |
| 58 | 79465 | ULBP3 | 1366 | -0.847 | -0.0631 | Yes |
| 59 | 5156 | PDGFRA | 1383 | -0.932 | -0.0466 | Yes |
| 60 | 4919 | ROR1 | 1385 | -0.954 | -0.0183 | Yes |
| 61 | 3329 | HSPD1 | 1402 | -1.183 | 0.0059 | Yes |
Table: GSEA details [plain text format]

  

Fig 2: GO\_CELL\_SURFACE      
 Blue-Pink O' Gram in the Space of the Analyzed GeneSet

  

Fig 3: GO\_CELL\_SURFACE: Random ES distribution      
 Gene set null distribution of ES for **GO\_CELL\_SURFACE**
